# Three-Dimensional Free Vibration Analysis of Thermally Loaded FGM Sandwich Plates

**DOI:** 10.3390/ma12152377

**Published:** 2019-07-25

**Authors:** Vyacheslav N. Burlayenko, Tomasz Sadowski, Svetlana Dimitrova

**Affiliations:** 1 Department of Applied Mathematics, National Technical University ’Kharkiv Polytechnic Institute’, 2 Kyrpychova Str., 61002 Kharkiv, Ukraine; 2 Department of Solid Mechanics, Lublin University of Technology, 40 Nadbystrzycka Str., 20-168 Lublin, Poland; 3 Department of Higher Mathematics, National Technical University ’Kharkiv Polytechnic Institute’, 2 Kyrpychova Str., 61002 Kharkiv, Ukraine

**Keywords:** functionally graded material, sandwich plates, free vibrations, 3D graded finite element, 3D thermoelastic finite element analysis

## Abstract

Using the finite element code ABAQUS and the user-defined material utilities UMAT and UMATHT, a solid brick graded finite element is developed for three-dimensional (3D) modeling of free vibrations of thermally loaded functionally gradient material (FGM) sandwich plates. The mechanical and thermal material properties of the FGM sandwich plates are assumed to vary gradually in the thickness direction, according to a power-law fraction distribution. Benchmark problems are firstly considered to assess the performance and accuracy of the proposed 3D graded finite element. Comparisons with the reference solutions revealed high efficiency and good capabilities of the developed element for the 3D simulations of thermomechanical and vibration responses of FGM sandwich plates. Some parametric studies are carried out for the frequency analysis by varying the volume fraction profile and the temperature distribution across the plate thickness.

## 1. Introduction

Sandwich panels are usually used instead of traditional structural elements made of metals and alloys, when increased strength and stiffness with little resultant weight are required for engineering applications [1,2,3]. Although sandwich panels provide outstanding structural features, this structural design has also drawbacks. A typical sandwich panel configuration has a high mismatch of material and geometrical properties between the face sheet and the core [4]. Due to this, a variation of the interfacial stresses induced by thermal or/and mechanical loads is significant at the face sheet–to-core interface [5,6,7]. Therefore, the performance and reliability of such tri-layer composites are eventually defined by the quality of the bonded interface [8,9]. When debonding arises between the skin and core material layers, sandwich panels significantly lose their load bearing capacity [10,11]. The modal dynamic characteristics of such panels damaged by debonding are changed [12,13,14,15] and their overall dynamic responses are modified [16,17,18,19] as well. Moreover, the debonding may cause eventual failure of the sandwich panels [20,21,22] under dynamic loads.

In regards to reducing or avoiding the debonding issue, functionally graded materials (FGMs), with mechanical and thermal properties that are smoothly distributed over the volume, have potential for use as basic layers in sandwich panels. Usually, such sandwich configuration is achieved by gradually changing the volume fraction of the FGM constituents across the sandwich plate thickness from the bottom face sheet to the top face sheet [23]. This removes the interface stress concentration and allows controlling deformation, dynamic responses, and etc, by customizing the gradation profile [24,25,26]. Another characteristic of FGM sandwich panels, with metal and ceramic material, is that they can be used at elevated service temperatures [27,28]. This fact, however, gives rise to a new demand for providing safe operation of FGM sandwich panels in thermal environments. Therefore, an accurate description of the thermomechanical behavior of FGM sandwich plates becomes mandatory, mainly, to prevent their thermal failure, as considered for FGM coatings in [29,30,31,32,33], amongst the most recent studies. At the same time, the analysis of free vibrations of FGM sandwich panels subjected to temperature, in the design stage, is important for estimating their overall performance.

To date, a considerable amount of research on the behavior of FGM plates and shells under temperature loading is found in the literature. Various analytical (or semi-analytical) and numerical methods have been developed for this, as recently reviewed by [34] reporting that there is intense research activity with respect to modeling thermally loaded FGM composites because reliable and practical analysis procedures, in terms of accuracy and computational efforts, are in high demand. In this regard, the development of two-dimensional (2D) models is motivated by computational efficiency, e.g., [35,36,37,38,39]. On the other hand, such models partially lose the accuracy of predictions due to simplifications caused by prescribing the behavior of shear deformations across the panel thickness. So-called quasi-three-dimensional theories, which adopt assumptions for both shear and normal deformations in the thickness direction, have recently been proposed as an improvement to 2D theories for more reliable analyses of FGM sandwich panels, for example, in [40,41,42]. Nevertheless, only three-dimensional (3D) models, which are not spoiled by any additional prescriptions inherent to 2D or quasi-3D theories, are able to address unique aspects stemming from the complex dynamic response of FGM sandwich panels [43]. However, there are two main obstacles to the use of 3D models for predictions of FGM plates. First, analytical exact 3D solutions for FGM sandwich plates are only available for simple cases of boundary conditions and geometries [44,45,46,47,48]. Secondly, although the finite element method (FEM), which is one of the leading computational tools, can solve problems associated with the complex geometry and different boundary conditions, the efficiency of conventional 3D finite element models is not suitable for real-scale FGM sandwich structures. The main reason for this is the layered approach for modeling material gradient with conventional finite elements because the conventional finite element has constant elastic properties over its domain. To overcome this obstacle inherent in models with conventional finite elements, the graded finite element, which assigns the material gradation profile at the element level, must be used instead. There are two techniques to elaborate a graded finite element. A nodal approximation of element material properties with interpolation functions identical to those utilized for the displacement field is used in the first technique, for example, as done in [49,50]. The second technique is based on sampling the material properties directly at the integration points of the element [51].

Not to mention fully coupled thermomechanical problems of FGM sandwich plates involving nonlinear material behaviors, the modal dynamics of FGM sandwich plates in thermal environment is strongly dependent on the distribution of thermal stresses across the plate thickness [52,53,54,55]. On the other hand, the thermomechanical behavior of such plates is defined by the material gradation profiles. Hence, a prerequisite for high-fidelity modeling is a precise thermoelastic analysis of such plates with accurately prescribed FGM properties. It is well-recognized that the FEM is a powerful means for solving multiphysical thermomechanical problems. Moreover, the method has been implemented in a series of commercially available codes, for instance, ABAQUS [56] which is popular among researchers and engineers. However, the package does not provide finite elements with a spatial variation of material properties. To accomplish this, programming of externally prescribed subroutines within the package environment is required. Some existing works suggest the use of the ABAQUS UEL subroutine which implements a material gradation in the FGM plates via a user-developed finite element, for example, [57]. Although this approach gives high flexibility in modeling, it requires the knowledge of an experienced user and extensive benchmarks for the performance of the element before simulations. Another approach for the implementation of varying material properties has been reported for ABAQUS 2D plane strain elements in [30,58,59], where the strain-stress state of FGM pavement and the thermomechanical behavior of the FGM plate have both been analyzed. The necessary material properties of studied functionally graded materials have been distributed at the Gauss points by coding appropriate material user-defined subroutines such as UMAT and UMATHT. In addition, this modeling technique has been extended to 2D plate/shell and 3D models of FGM plates in [60,61], respectively, however, only the static bending analysis has been simulated there. Recently, three-dimensional finite element models of FGM sandwich plates for dynamic modal analyses have been developed in [62,63].

The aim of this study is to propose an efficient approach for implementing a 3D graded finite element into ABAQUS code to perform a computationally accurate free vibration analysis of thermally loaded FGM sandwich plates. A novel graded finite element has been developed based on the 3D brick graded finite element proposed for the free vibration analysis of 3D FGM sandwich plates in [62,63]. In our study, we extend the functionality of the element to use it for modeling FGM sandwich plates under thermal loading. First, we consider a thermomechanical analysis to compute through-the-thickness distributions of displacements and stresses in the sandwich plates. Then, with a known thermally induced stress state, the free vibration analysis is carried out. The 3D brick graded element has been developed by coding a combination of the subroutines such as UMAT, UMATHT, and USDFLD similar to the 2D thermomechanical finite element analysis presented in [59]. The performance of the proposed 3D graded element has been demonstrated by the 3D modelling of heat transfer and free vibrations of FGM sandwich plates subjected to thermal loading. The accuracy of the graded element has been validated by comparison with results available in the literature for FGM sandwich plates. Parametric studies have also been carried out to determine the effect of varying volume fraction profiles and the temperature on natural frequencies and associated mode shapes. We believe that the results of this research can be used as a benchmarks for 2D solutions and results obtained by other numerical methods.

## 2. Problem Formulation

For the sake of completeness, the thermomechanical problem for a continuum made of a functionally graded material is briefly summarized in this section. Throughout the section we adopt the usual notations used in most books on continuum mechanics, which can be referred to for more details.

### 2.1. Thermomechanical Problem

**Figure 1:**
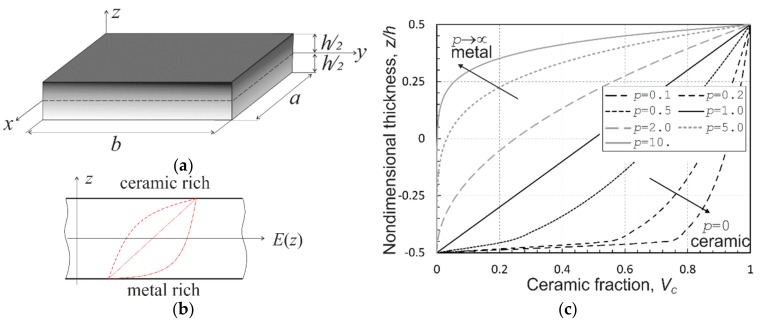
Sketches of: (**a**) functionally gradient material (FGM) sandwich panel; (**b**) through-the-thickness gradations of material properties; and (**c**) variations of ceramic volume fraction variations for various power-law indexes *p*.

Let us consider the FGM sandwich plate as a 3D deformable medium occupying the domain $\Omega \in \left[0,a\right]\times \left[0,b\right]\times \left[-\frac{h}{2},+\frac{h}{2}\right]$ bonded by the surface, ∂Ω⊂Ω, at an instant of time, $t\in \left[0,t_{end}\right]$ The plate is defined at a given temperature $T_{0}$ in the unstressed reference configuration with respect to a rectangular Cartesian co-ordinate system, $x_{i}=\left(x,y,z\right)$, with the *z*-axis aligned along the plate thickness and with the plane, z=0, coinciding with the mid-plane of the sandwich plate. In addition, the planes z=±h/2 refer to the bottom $\partial \Omega^{-}$ and the top $\partial \Omega^{+}$ plate surfaces, respectively, where $\partial \Omega \backslash \partial \Omega^{-}\cup \partial \Omega^{+}=\left[-\frac{h}{2},+\frac{h}{2}\right]$, as shown in Figure 1a.

In the Lagrangian description, the equations of mechanical motion and thermal equilibrium at each spatial point, x, of the domain, Ω, at a time instant, t, in the absent of body forces and internal heat sources can be presented as [64]:

(1) $$\begin{array}{c} \nabla \cdot \sigma =\rho \left(x\right)\ddot{u},\\ -\nabla \cdot q=\rho \left(x\right)c\left(x\right)\dot{\theta }+\beta \left(x\right)T_{0}\mathrm{Tr}\left(\dot{\varepsilon }\right) \end{array}$$

where, σ and **q** are the Cauchy stress tensor and the heat flux vector, respectively; **u** is a displacement field and *θ* stands for a temperature field associated with a change of the instantaneous temperature $T\left(x,t\right)$ above the reference temperature $T_{0}$ at time $t\in \left[0,t_{end}\right]$; and $\rho \left(x\right)$, $c\left(x\right)$, and $\beta \left(x\right)$ denote the mass density, the specific heat, and the stress-temperature modulus, respectively, which are functions of a spatial position x.

Assuming small displacements and deformations, the infinitesimal strain tensor as a sum of elastic mechanical “*el*” and thermal “*th*” parts is expressed by

(2) $$\begin{array}{c} \varepsilon =\varepsilon^{el}+\varepsilon^{th}=\frac{1}{2}\left(\nabla u+{\left(\nabla u\right)}^{T}\right),\\ \varepsilon^{th}=\alpha \left(x\right)\theta I, \end{array}$$

where, $\alpha \left(x\right)$ is the coefficient of thermal expansion and **I** is the identity tensor.

Consider the FGM is a linear isotropic material that complies with the classical law of thermal conductivity. Then, the thermoelastic constitutive equations of the FGM sandwich plate have a form:

(3) $$\begin{array}{c} \sigma =\lambda \left(x\right)\mathrm{Tr}\left(\varepsilon \right)I+2\mu \left(x\right)\varepsilon -\beta \left(x\right)\theta I=D\left(x\right):\varepsilon -\beta \left(x\right)\theta I,\\ q=-\kappa \left(x\right)\nabla \theta , \end{array}$$

where, the Lamé constants $\lambda \left(x\right)$ and $\mu \left(x\right)$ of the elasticity tensor $D\left(x\right)$, the modulus $\beta \left(x\right)=\alpha \left(3\lambda +2\mu \right)$ and the conductivity $\kappa \left(x\right)$ are pointwise functions of location.

Two types of the boundary conditions must be specified on the plate surface ∂Ω at any instant in time. The mechanical boundary conditions are prescribed by displacements $\;\bar{u}$ on the boundary $\partial \Omega_{u}$ and traction $\bar{t}$ on the boundary $\partial \Omega_{t}$, where, $\partial \Omega_{u}\cup \partial \Omega_{t}=\partial \Omega$. In a similar manner, the thermal boundary conditions are defined by a prescribed temperature $\bar{T}$ and heat flux ${\bar{q}}_{1}$ and/or an exposure to an ambient temperature through convection so that ${\bar{q}}_{2}=\hat{h}\left(x\right)\left(T-T_{\infty }\right)$ on the plate surfaces $\partial \Omega_{\theta }$ and $\partial \Omega_{q}=\partial \Omega_{q_{1}}\cup \partial \Omega_{q_{2}}$, respectively. Here, $\hat{h}\left(x\right)$ is the heat film transfer coefficient and $T_{\infty }$ is the temperature of the surrounding medium [64].

Using the principle of virtual work and collecting (1) to (3) with appropriate boundary conditions, the system of mechanical and energy equations can be rewritten in the weak form as follows:

(4) $$\begin{array}{c} \int_{\Omega }^{}\left\{\sigma :\nabla \delta u+\rho \left(x\right)\ddot{u}\cdot \delta u\right\}dV-\int_{\partial \Omega_{t}}^{}\bar{t}\cdot \delta udA=0,\\ \int_{\Omega }^{}\left\{\left[\rho \left(x\right)c\left(x\right)\dot{\theta }+T_{0}\beta \left(x\right)\mathrm{Tr}\left(\varepsilon \right)\right]\delta \theta -q\cdot \nabla \delta \theta \right\}dV+\int_{\partial \Omega_{q}}^{}\bar{q}\delta \theta dA=0, \end{array}$$

for all kinematically admissible virtual displacement δu and temperature δθ fields.

### 2.2. Properties of FGM

We assume that the sandwich plate is made of a two-phase metal-ceramic functionally graded material and, also, without loss of generality, a smooth variation of material thermomechanical properties across the plate thickness only, i.e., in the *z*-direction, see Figure 1b. Herewith, it is deemed that the face sheets (or skins) are homogeneous, i.e., pure metal on one side and pure ceramic on the other one, with a small or negligible small thickness as compared with the thick metal-ceramic FGM core. In addition, we suggest that the gradation profile of the ceramic volume fraction from the bottom to top sandwich plate skins is known and is determined by a power-law function in the form:

(5) $$V_{c}=V_{c}^{-}+\left(V_{c}^{+}-V_{c}^{-}\right){\left(\frac{1}{2}+\frac{z}{h}\right)}^{p}$$

where, $V_{c}^{-}$ and $V_{c}^{+}$ are the volume fraction of ceramic on the bottom and top surfaces, respectively. The case of $V_{c}^{-}=0$ and $V_{c}^{+}=1$ refers to the gradation profile from pure metal on z=−h/2 to pure ceramic on z=+h/2. It follows from (5) that the FGM plate is ceramic-rich when the parameter p<1, and metal-rich when the parameter p>1. Figure 1c shows the volume fraction variation of the ceramic phase along the plate thickness depending on the values of the power-law index *p*.

The effective mass density, and the thermal and mechanical properties at a point of the FGM are specified based on the “rule of mixture” as follows:

(6) $$P\left(z\right)=P_{m}+\left(P_{c}-P_{m}\right)V_{c}$$

where, *P*(*z*) represents either the mass density or any of the thermomechanical parameters; and the subscripts “*m*” and “*c*” are the metallic and ceramic phases whose volume fractions are such that $V_{c}+V_{m}=1$.

In turn, each of the parameters may depend on the temperature in the form:

(7) $$P\left(T\right)=P_{0}\left(P_{-1}\frac{1}{T}+1+P_{1}T+P_{2}T^{2}+P_{3}T^{3}\right)$$

where, $P_{0}$ stands for material parameters at the reference temperature $T_{0}$ and $P_{-1}$, $P_{1}$, $P_{2}$, and $P_{3}$ are constants specifying the temperature dependence of the material at the instantaneous temperature *T*. 

Finally, since the gradient in properties occurs only along the plate thickness direction, then the material tensor in (3) in the Voigt notation is as follows:

(8) $$D\left(z\right)=\left[\begin{array}{cc} \begin{array}{ccc} 2\mu \left(z\right)+\lambda \left(z\right) & \lambda \left(z\right) & \lambda \left(z\right)\\ \lambda \left(z\right) & 2\mu \left(z\right)+\lambda \left(z\right) & \lambda \left(z\right)\\ \lambda \left(z\right) & \lambda \left(z\right) & 2\mu \left(z\right)+\lambda \left(z\right) \end{array} & 0\\ 0 & \begin{array}{ccc} \mu \left(z\right) & & \\ & \mu \left(z\right) & \\ & & \mu \left(z\right) \end{array} \end{array}\right]$$

## 3. Method of Solution

A displacement-based FEM framework is used for solving the problem formulated in Section 2.

### 3.1. Finite Element Discretization

In the context of FEM, the actual continuous model of the sandwich plate is idealized by an assemblage of arbitrary non-overlapping finite elements, $\Omega =U_{e=1}^{N}\Omega^{e}$, interconnected at nodal points. In each base element the displacement vector, **u**^(*e*^^)^, and a scalar function of temperature, *θ*^(*e*^^)^, are approximated by suitable interpolation functions such that

(9) $$\begin{array}{c} u^{\left(e\right)}\left(x,t\right)=N\left(x\right)U^{\left(e\right)}\left(t\right),\\ \theta^{\left(e\right)}\left(x,t\right)=\tilde{N}\left(x\right)\Theta^{\left(e\right)}\left(t\right) \end{array}$$

Here, the summation over all nodal points of the base element is intended, also, $N=\left[N_{I}\left(x\right)\right]$ and $\tilde{N}=\left[N_{P}\left(x\right)\right]$ are matrices of the shape functions $N_{I}$ and $N_{P}$ for the displacements and the temperature, respectively, associated with certain nodes *I* and *P*. The vectors $U^{\left(e\right)}$ and $\Theta^{\left(e\right)}$ are the nodal unknown displacements and temperature at those nodes.

The coupling between the mechanical and thermal problems is assumed to be due to temperature only, i.e., there is no feedback on the energy expression through the displacement field. This assumption is reasonable if a thermomechanical model is used that does not involve internal variables, such as plastic strains for computing the energy dissipation rate [56]. Substituting the displacement and temperature approximations (9) into the variational equalities (4) and accounting for the material laws in (3), we arrive at the system of semidiscrete equations of a one-way thermomechanical problem at the element level as follows:

(10) $$\left[\begin{array}{cc} M^{\left(e\right)} & 0\\ 0 & 0 \end{array}\right]\left\{\begin{array}{c} {\ddot{U}}^{\left(e\right)}\\ {\ddot{\Theta }}^{\left(e\right)} \end{array}\right\}+\left[\begin{array}{cc} 0 & 0\\ 0 & C^{\left(e\right)} \end{array}\right]\left\{\begin{array}{c} {\dot{U}}^{\left(e\right)}\\ {\;\dot{\Theta }}^{\left(e\right)} \end{array}\right\}+\left[\begin{array}{cc} K_{u}^{\left(e\right)} & K_{u\theta }^{\left(e\right)}\\ 0 & K_{\theta }^{\left(e\right)} \end{array}\right]\left\{\begin{array}{c} U^{\left(e\right)}\\ \Theta^{\left(e\right)} \end{array}\right\}=\left\{\begin{array}{c} F_{u}^{\left(e\right)}\\ F_{\theta }^{\left(e\right)} \end{array}\right\}$$

Forms of the element matrices involved in (10) are found, for example, in [59]. The assembly operation $\left(•\right)=A_{e=1}^{N}{\left(\circ \right)}^{\left(e\right)}$ over all the finite elements leads to the global system of semidiscrete equations for the thermoelastic problem in the form:

(11) $$\left[\begin{array}{cc} M & 0\\ 0 & 0 \end{array}\right]\left\{\begin{array}{c} \ddot{U}\\ \ddot{\Theta } \end{array}\right\}+\left[\begin{array}{cc} 0 & 0\\ 0 & C \end{array}\right]\left\{\begin{array}{c} \dot{U}\\ \;\dot{\Theta } \end{array}\right\}+\left[\begin{array}{cc} K_{u} & K_{u\theta }\\ 0 & K_{\theta } \end{array}\right]\left\{\begin{array}{c} U\\ \Theta \end{array}\right\}=\left\{\begin{array}{c} F_{u}\\ F_{\theta } \end{array}\right\}$$

where, M, $K_{u}$, $K_{\theta }$ and C are the usual global mass, stiffness, conductivity, and capacity matrices, respectively; $K_{u\theta }$ is the coupling thermoelasticity matrix; U and Θ are the global vectors of nodal displacements and nodal temperature, the dots over them correspond to the time derivatives of these vectors; and $F_{u}$ and $F_{\theta }$ are global vectors of the mechanical and thermal forces.

In the case of an uncoupled formulation, the temperature is given as an external load and it is not a primary variable in the mechanical analysis. More specifically, the thermal virtual work leads to the initial stress matrix known as a geometric stiffness matrix $K_{G}$, which contains the terms due to the temperature loading on the leading diagonal. Thus, first, the temperature field is computed at the given thermal and displacement boundary conditions. Then, a temperature profile, known for solving the mechanical problem with the same displacement boundary conditions, is used to find the displacement and stress fields. The nonlinear finite element equations of the thermomechanical problem are solved by an iterative method, where the nonlinear terms of linearized equations are evaluated as a known solution from the preceding iteration. The Newton-Raphson iterative method is used in ABAQUS [56]. Finally, the frequency analysis, which accounts for the initial deformed state associated with the temperature-induced stresses is carried out by extracting natural frequencies from the eigenvalue-type equation:

(12) $$\left(\left(K+K_{G}\right)-\omega^{2}M\right)\phi =0$$

where, *ω* is an undamped circular frequency and ϕ is a vector associated with mode shape at a specific frequency *ω*.

### 3.2. Three-Dimensional Graded Element

The thermomechanical analysis and the modal frequency extraction procedure for FGM sandwich plates are carried out with the ABAQUS/Standard code using three-dimensional models. Since conventional 3D finite elements, which are available in the ABAQUS finite element library, are not able to model a variation of the thermal and mechanical properties within the element volume, a 3D graded finite element incorporating gradients of material properties is developed.

**Figure 2:**
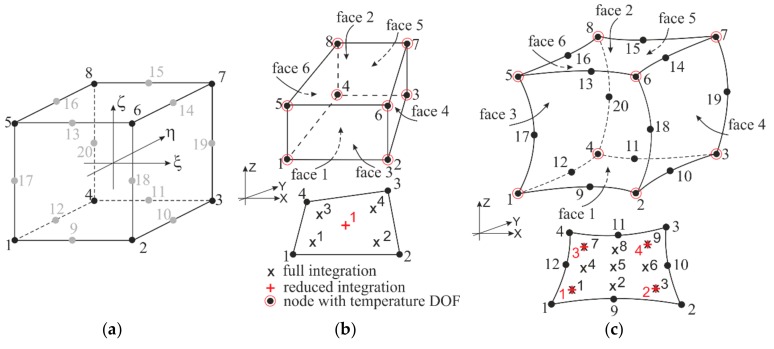
Isoparametric 3D brick finite elements from [56]: (**a**) master element; (**b**) eight-node linear element with reduced and full integration schemes^1^; and (**c**) 20-node quadratic element with reduced and full integration schemes^1^. ^1^ Numbering of integration points for output is shown in the element layer closest to the face 1, and the integration points in the other layers are numbered consecutively.

As mentioned in the Introduction, a 3D graded finite element has been developed for performing the modal frequency analysis of FGM sandwich plates in [62,63]. To incorporate a variation in the elastic properties of the heterogeneous material into the finite element, the material user-defined subroutine UMAT that establishes the tangent element stiffness matrix was programmed, while the average mass density value was adopted to determine the element mass matrix. In our study, the performance of the 3D graded finite element is extended to provide thermal loading and temperature-dependent material properties for carrying out the thermomechanical analysis of the FGM sandwich plates. Such an analysis requires computing and storing the internal thermal energy and the heat flux which comply with the energy balance equation (1) and Fourier’s law of heat conduction (3), respectively, as well as modifying the mechanical behavior by accounting for thermal strains (2). The implementation of a spatial variation of the thermal and modified mechanical properties in the selected direction in the 3D graded finite element has been done following the procedure outlined in [59] for a 2D graded finite element. With this approach, a master 3D temperature-displacement finite element, either eight-node linear C3D8 or twenty-node quadratic C3D20 brick isoparametric element with either reduced or fully integration scheme, which are available in ABAQUS (Figure 2), has been supplemented by a combination of user-defined subroutines such as UMAT, UMATHT, and USDFLD, [56]. In addition, it should be mentioned that ABAQUS interpolates the temperature field using only the first-order approximation regardless of the order of approximation of the displacement field, as shown in Figure 2b,c.

The material subroutine UMAT was programmed to define a through-the-thickness variation of the Lamé constants, $\lambda \left(x\right)$ and $µ\left(x\right)$, and the stress-temperature modulus, $\beta \left(x\right)$, associated with the thermal expansion coefficient, $\alpha \left(x\right)$. The distributions across the thickness of the material thermal properties were incorporated into the element by coding the specific heat, $c\left(x\right)$, the thermal conductivity, $\kappa \left(x\right)$, and the film heat transfer, $\hat{h}\left(x\right)$, coefficients in the UMATHT subroutine. Finally, the through-the-thickness variation of mass density was incorporated into the element using the USDFLD subroutine. Moreover, if the material parameters were deemed to exhibit a temperature dependence, appropriate relationships (7) with given coefficients for each material parameter were also programmed as functions of the temperature in the mentioned subroutines. In doing so, the instantaneous temperature, *T*, was known as it is a variable passed for information at each time increment in the subroutines, and therefore, the property was able to be computed at any current temperature value.

By running the ABAQUS code, the element matrices, presented in (10), which involve variations of the material thermal and mechanical properties coded in accordance with a certain relation for FGM constituents, have been generated by calling the corresponding user-defined subroutines. Thus, the material properties that account for the given material gradation profiles have been assigned directly at the Gauss integration points of the element (see Figure 2b,c). In such a way, any arbitrary material gradient, for example, a power-law distribution of the ceramic phase in the thickness direction of the plate can be prescribed. More details of the implementation of graded elements into the ABAQUS code are found in [30,59] and the ABAQUS manual [56]. In addition, it is important to note that the mass density, averaged over the FGM sandwich plate volume [62,63], was used in the free vibration analysis instead of its spatial representation in the case of thermomechanical analysis.

## 4. Comparison Study

The performance of the 3D graded element described above for solving thermomechanical and free vibration problems has been verified by comparing calculated numerical solutions with results available in the literature.

### 4.1. Cube Problem

**Figure 3:**
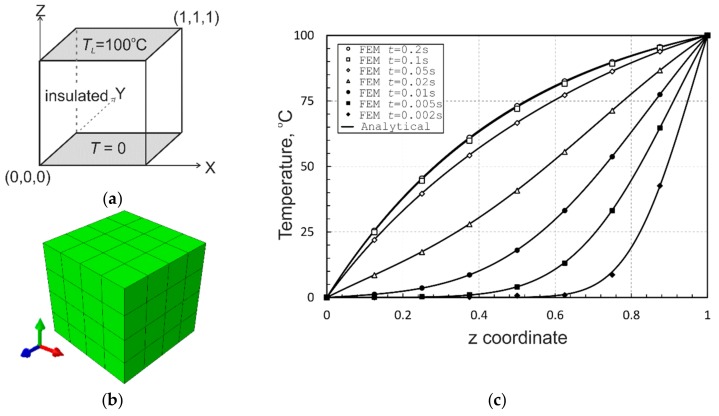
The FGM unit cube problem: (**a**) geometry and thermal boundary conditions; (**b**) finite element mesh; and (**c**) transient temperature profiles.

As a first validation problem, a unit FGM cube (*L* = 1) that has been subjected to prescribed temperatures on two opposite sides and insulated in all the other sides is considered, Figure 3a. This problem has been solved analytically and numerically using the boundary element method in [65]. The top surface of the cube *z* = 1 is maintained at the temperature *T_L_* = 100 °C, while the temperature of the bottom surface at *z* = 0 is 0 °C. The reference temperature is also assumed to be 0 °C. The variations of thermal conductivity and specific heat along the *z*-axis are defined by the expressions:

(13) $$\kappa \left(z\right)=\kappa_{0}e^{2\zeta z}=5e^{2z}$$

and

(14) $$c\left(z\right)=c_{0}e^{2\zeta z}=e^{2z}$$

The analytical solution for the temperature in the transient thermal analysis is known [65] as

(15) $$\theta \left(z,t\right)=T_{L}\frac{1-e^{-2\zeta z}}{1-e^{-2\zeta L}}+\overset{\infty }{\underset{n=1}{\sum }}B_{n}\sin\left(\frac{\pi nz}{L}\right)e^{-\zeta z}e^{-\left(\frac{\pi^{2}n^{2}}{L^{2}}+\zeta^{2}\right)\epsilon t}$$

where, the coefficients, $B_{n}$, are given in the form:

(16) $$B_{n}=-T_{L}\frac{2e^{\zeta L}}{\zeta^{2}L^{2}+\pi^{2}n^{2}}\left[\zeta L\sin\left(\pi n\right)\frac{1+e^{-2\zeta L}}{1-e^{-2\zeta L}}-\pi n\cos\left(\pi n\right)\right]\;\mathrm{and}\;\epsilon =\kappa_{0}/c_{0}$$

In the finite element simulations, the cube is discretized with 4 × 4 × 4 twenty-node quadratic brick graded elements, as illustrated by Figure 3b. A transient heat transfer analysis is performed with ABAQUS, calling the material subroutines UMATHT and USDFLD. The temperature profile along the *z*−axis is plotted at different times for the given exponential material variations and compared with the analytical solutions, as shown in Figure 3c. It is evident from the plot that the numerical and analytical results are in excellent agreement.

### 4.2. Analysis of FGM Plates

**Figure 4:**
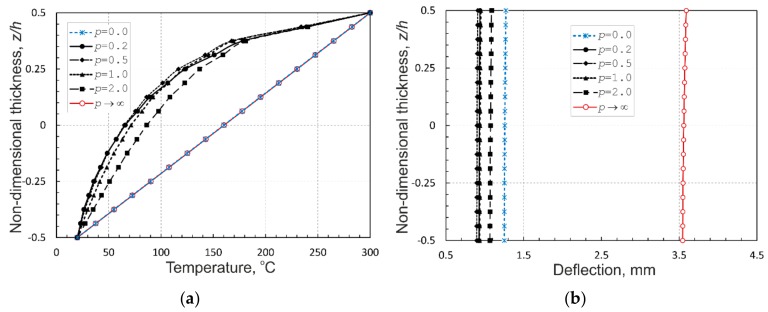

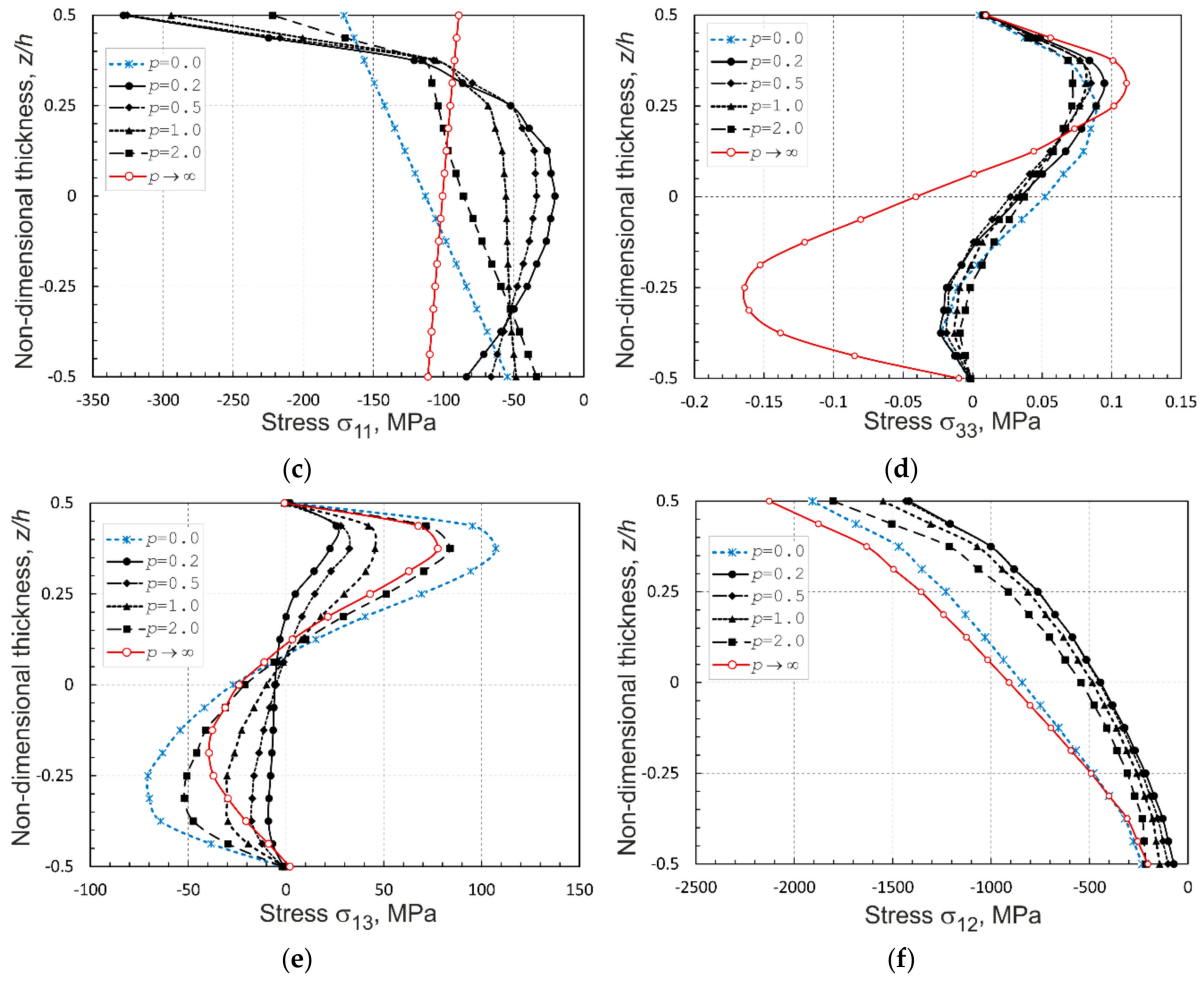
Through-the-thickness profiles of: (**a**) temperature at $\left(a/2,\;b/2,z\right)$; (**b**) deflection at $\left(a/2,\;b/2,z\right)$; (**c**) longitudinal normal stress $\sigma_{11}$ at $\left(a/2,\;b/2,z\right)$; (**d**) transverse normal stress $\sigma_{33}$ at $\left(a/2,\;b/2,z\right)$; (**e**) transverse shear stress $\sigma_{13}$ at $\left(0,\;b/2,z\right)$; and (**f**) in-plane shear stress $\sigma_{12}$ at $\left(0,\;0,z\right)$ in the simply supported aluminum-zirconia FG square plate.

As a second example, an aluminum-zirconia functionally graded square plate with sides *a* = *b* = 0.2 m and thickness *h* = 0.01 m, as studied in [35] is considered. The plate is assumed to be simply supported on all the edges and is exposed to a temperature field such that the ceramic-rich top surface is held at 300 °C and the metal-rich bottom surface is held at 20 °C. A stress-free state is assumed to be at 0 °C. The material thermomechanical parameters of the FGM plate are listed in Table 1. For the purpose of comparison, the values of the volume fraction exponent, *p*, in (5) have been accepted as 0.0 (pure ceramic), 0.2, 0.5, 1.0, 2.0 and ∞ (pure metal). Figure 4a shows the variation of the temperature through-the-thickness profiles of the FGM plate depending on the exponent values. By comparing the temperature profiles shown in Figure 4a with those illustrated in [35] (p. 680), one can conclude that the plots are nearly identical. 

With these calculations it was found that the calculated results converge to the reference data in [35] with an increase of the number of graded elements in the thickness direction. The best agreement between both the solutions was achieved using eight graded elements across the plate thickness. This resulted in time-consuming computations in the case of cubic elements used in the mesh. In order to speed up the computations, brick graded elements, with an aspect ratio 4:4:1, have been used instead. The elements provided more than 10 times faster computations with the same through-the-thickness profiles for variations of temperature and quite acceptable results for temperature-induced deflections and stresses across the thickness, as illustrated in Figure 4b–f, respectively, as compared with those in [44]. Therefore, such elements are used in the calculations to follow.

As shown in Figure 4a, it is obvious that the metal and ceramic plates have linear temperature variations through-the-thickness profiles, while the FGM plates possess nonlinear temperature profiles with much lower temperatures in the bottom part of the plate thickness due to the insulation effect of ceramic located over the metallic part. Nevertheless, although FGM plates have intermediate properties between the pure ceramic and metal plates, their central deflections do not show intermediate values between those of the homogeneous plates, as seen in Figure 4b. This is related to the fact that the deflection depends on the product of the temperature and the thermal expansion coefficient. The latter is larger in the metal-rich region, while the temperature is higher in the ceramic-rich portion. As a result, the thermal strains are not uniform over the plate thickness and the responses of the FGM plates are not intermediate to those of the pure metal and ceramic plates. The temperature-induced through-the-thickness distributions of central longitudinal and transverse normal stresses, and a transverse shear stress at the center of plate edge and an in-plane shear stress at the plate corner, shown in Figure 4c–f, respectively, demonstrate that the longitudinal normal stresses in the FGM plates exhibit nonlinear profiles in contrast to linear ones in the pure metal and ceramic plates, whereas, the transverse normal stress and the shear stresses of all the plates have rather similar profiles and, in the case of FGM plates, the stress distributions crucially depend on the power-law index *p*.

In order to evaluate the accuracy of the developed graded element in the free vibration analysis of thermally loaded FGM plates, the natural frequencies of a fully clamped (CCCC) square FGM plate with the thickness-side ratio h/a=0.1 have been computed and compared with those available in [46]. Steel-silicon SUS304/Si_3_Ni_4_ functionally graded plates were considered. The properties of constituents of the FGM are given in Table 1, while the temperature-dependent constants of the constituents can be found in [46] (p. 737, Table 1). The FGM plates were assumed to be subjected to different temperatures equal to 300 K, 600 K and 800 K which are uniformly distributed across the plate thickness. The natural frequencies of the FGM plates extracted from the finite element analysis have been nondimensionalized as follows:

(17) $$\bar{\omega }=\frac{\omega a^{2}}{\pi^{2}}\sqrt{\frac{I_{m}}{D_{m}}}$$

where, $I_{m}=h\rho_{m}$ and $D_{m}\;=\;E_{m}h^{3}/\left(12\left(1-\nu_{m}^{2}\right)\right)$ are expressed using the appropriate values of the stainless steel at the reference temperature $T_{0}$ = 300 K. Table 2 shows a good agreement between the computed nondimensional frequencies ($\omega_{FEM}$) and the results ($\omega_{Ref.}$) reported in [46].

## 5. Parametric Study

After establishing the correctness of the developed 3D graded finite element, parametric studies are performed to investigate the effects of gradation profiles in thermo-elastic properties and temperature distributions on the free vibrations of FGM sandwich plates.

First, we consider the free vibration analysis of SUS304/Si_3_Ni_4_ functionally graded square sandwich plates with the skins’ thickness negligible as compared with the core thickness. The geometry and material properties of the sandwich plates were identical to the analysis for the FGM plate in Section 4.2. It is assumed that across the thickness, the sandwich plates may be subjected to either a uniform temperature field, $T_{b}=T_{t}=T$, or a temperature profile associated with a steady-state heat transfer due to differently prescribed temperatures on the bottom, $T_{b}$, and top, $T_{t}$, plate surfaces. Two types of boundary conditions, i.e., all edges simply supported (SSSS) and all edges clamped are used in the calculations. Five different material gradations defined by the power-law index *p* = 0.2, 0.5, 1, 5 and 10 as well as pure ceramic (p=0) and metal (p→∞) homogeneous plates are examined. In Table 3 and Table 4, the first ten nondimensional natural frequencies $\bar{\omega }$ of simply supported and clamped FGM plates under the uniform temperature of *T*=600 K are presented, respectively. In addition, in Table 3 and Table 4, the frequencies calculated with the 3D graded elements are compared with those available in [46] for the studied FGM plates.

**Figure 5:**
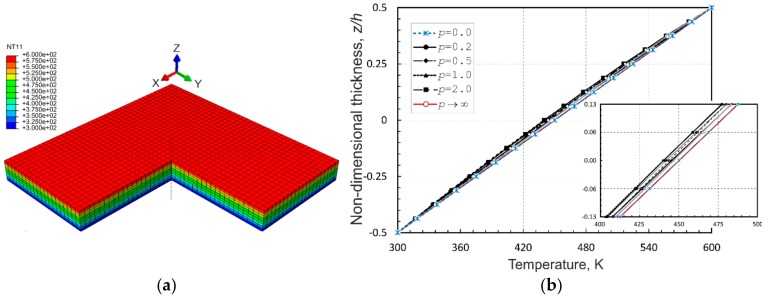
For simply supported and clamped plates: **(a)** temperature distribution and **(b)** through-the-thickness profiles of the temperature.

Similar to the previous study, the first ten nondimensional natural frequencies of simply supported and clamped FGM plates subjected to the temperature profiles following from the solution of the thermomechanical analysis under steady-state conditions with the prescribed temperature on the top (ceramic) surface $T_{t}=$ 600 K and at the reference temperature on the bottom (metal) surface $T_{b}=T_{0}=\;$300 K are collected in Table 5 and Table 6, respectively. The contour plot of the temperature distribution within the plate (a quarter of plate is removed from the presentation to illustrate the temperature distribution inside the plate) and the variations of temperature across the thickness (at the central section of the plate) depending on the power-law index *p*, which have been predicted by the thermomechanical analysis for the SSSS and CCCC plates, are illustrated in Figure 5a,b. It is evident from the latter plot that the effect of *p* is not significant for this material and the nonlinear temperature profiles are very close to the linear temperature distribution.

**Figure 6:**
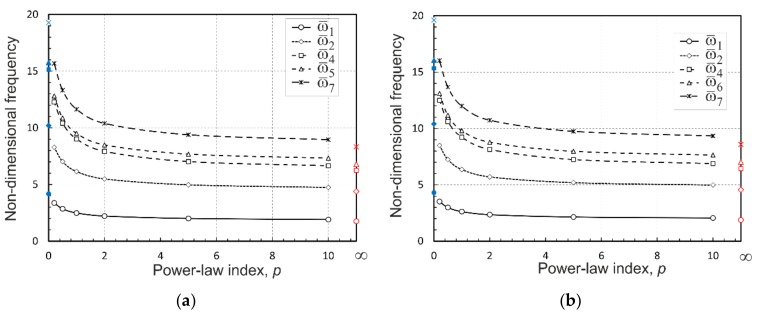
The effect of the material gradation profile on natural frequencies of: (**a**) SSSS plates and (**b**) CCCC plates.

It is worth noting that there is very good agreement between the present results and the referenced solutions, as seen in Table 3 to Table 6. This demonstrates the accuracy and effectiveness of the 3D graded finite element developed in the present work. In addition, for the sake of clear demonstration of the effect of the material gradation profile on the natural frequencies, some frequencies from Table 5 and Table 6 for the simply supported and clamped FGM plates subjected to the nonlinear temperature rise are plotted as functions of the volume fraction exponent *p* in Figure 6a,b, respectively. It is obvious from the plots that the frequencies decrease with an increasing in the power-law index, i.e., growing the percentage of ceramic fraction in the top thickness of the FGM plates. In doing so, the higher frequencies show more intensive descending trends.

Next, sandwich plates with thickness of skins $h_{f}=0.1h$ and thickness of core $h_{c}=0.8h$, which are referred to as 1-8-1 sandwich configurations, are considered for the free vibration analysis. Three different boundary conditions, such as fully simply supported, fully clamped, and two edges simply supported and two edges clamped (SCSC) are examined. It is assumed that the sandwich plates have homogenous skins such that the top and bottom skins are pure ceramic and pure metal, respectively, and the core is SUS304/Si_3_Ni_4_ functionally graded material identical to that in the previous study with the volume fraction exponent *p* = 0.2, 0.5, 2 and 10. The thermomechanical constants of the material constituents are listed in Table 1. The sandwich plates are assumed to be subjected to the prescribed temperature on the top surface $T_{t}$, and the bottom surface is at the reference temperature, i.e., $T_{b}=\;T_{0}$. For the free vibration analyses, first, temperature gradients across the thickness of the plates are computed using the thermomechanical analysis under steady-state conditions. Three different temperatures $T_{t}=$ 300, 400 and 600 K are applied to the ceramic surface, while the metal surface is kept constant and equal to the reference temperature $T_{0}$ = 300 K.

The nondimensional natural frequencies $\tilde{\omega }=\omega \left(a^{2}/h\right)\sqrt{\rho_{m}/E_{m}}$ of the FGM sandwich plates for SSSS, CCCC and SCSC boundary conditions, subjected to different temperature profiles following from the solution of the thermo-mechanical analysis and for different material gradients defined by the power-law index are tabulated in Table 7, Table 8 and Table 9, respectively. In Table 7, Table 8 and Table 9, for the sake of controlling the accuracy of simulations, the first two frequencies have been compared with those presented in [39].

**Figure 7:**
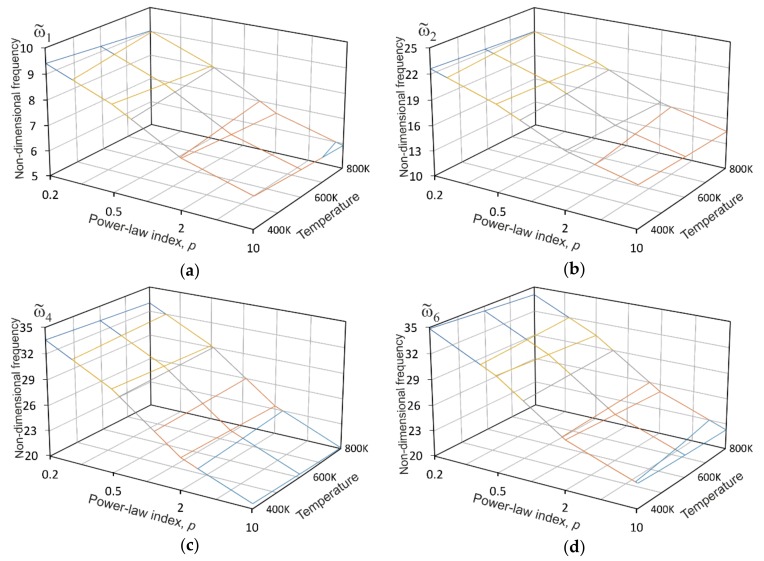
The effect of the temperature on natural frequencies of SSSS square 1-8-1 sandwich plates with different material gradients in SUS304/Si_3_Ni_4_ cores: (**a**) the first nondimensional frequency; (**b**) the second non-dimensional frequency; (**c**) the fourth non-dimensional frequency; and (**d**) the sixth non-dimensional frequency.

By inspecting Table 7 to Table 9, one can observe that the first two frequencies obtained from the present finite element model involving the 3D graded element are in a good agreement with the finite element results reported in [39] for all the thermal and displacement boundary conditions considered in the calculations. In addition, to show the effect of temperature on the natural frequencies of the SSSS sandwich plates with different material gradients in SUS304/Si_3_Ni_4_ cores, several nondimensional frequencies from Table 7 are presented as a two-dimensional function of the temperature and the power-law index in Figure 7. It is clearly seen from the plots that all the frequencies decrease with increasing temperature for each value of *p*, in other words, as expected, the FGM sandwich plates become more compliant because of the decrease in material stiffness at higher temperatures. Here, the variation of the volume fraction exponent affects the natural frequencies to a greater extent than the temperature in the considered range. It is also important to mention that the natural frequencies of the CCCC and SCSC sandwich plates exhibit similar responses with rising temperature, and by increasing the power-law index.

## 6. Conclusions

The free vibrations of FGM sandwich plates under temperature loading conditions are examined. The natural frequencies of thermally loaded FGM plates are computed using a model based on the 3D graded finite element developed within the ABAQUS code environment. The material gradient was assumed to vary in the thickness direction of the plates according to a power-law distribution of the volume fractions. The rule of mixture was used to evaluate the effective material properties of the FGM. The FGM was implemented into the conventional 3-D elements of ABAQUS code via a combination of user-defined subroutines such as UMAT, UMATHT, and USDFLD (the codes can be downloaded from http://polonez.pollub.pl/deliverables/). In the simulations of FGM sandwich plates, the thermomechanical analysis, used to obtain a temperature profile and associated with temperature-induced displacement and stress fields, couples with a frequency analysis to calculate natural frequencies and mode shapes accounting for a temperature-defined base state. The latter analysis adopted the average mass density instead of its spatial distribution adopted by the former one. The convergence analysis of the present FE model has been done to validate the accuracy of the numerical results by comparing them with the solutions previously reported in the literature and to estimate the model computational efficiency. The effects of different thermal loads, imposed at the external surfaces in steady-state conditions, and different displacement boundary conditions and material parameters, associated with a variety of volume fractions of the material constituents, on the frequencies of FGM sandwich plates are discussed in detail. The following conclusions can be drawn from the present study:

• The use of the graded finite elements for analyzing FGM sandwich plates provides a more efficient modeling approach than homogeneous elements to achieve high-fidelity results.
• The solutions of thermomechanical analysis reveal that a temperature profile calculated with the 3D model is important to predict correct thermal-induced displacement and stress distributions, which, in turn, affect the accuracy of calculated natural frequencies and mode shapes of thermally loaded FGM plates. The thermomechanical analysis also permits the mode shapes to be analyzed in terms of the temperature and stresses.
• It is observed from the simulations that the natural frequencies decrease as the volume fraction of ceramic decreases across the thickness of the FGM plates.
• The natural frequencies have a tendency to decrease with an increase of temperature for each of the functionally graded material profiles studied.
• This work forms a convenient tool for subsequent dynamic analyses of FGM sandwich plates under different temperature conditions with accurate finite element solutions provided by the ABAQUS commercial code.

The developed graded finite element can also be adopted to a 3D crack sensitivity analysis associated with nondestructive testing of FGM sandwich panels and welding-adhesive joints as proposed in [66,67]. This will be a subject of our future research.

## Figures and Tables

**Table 1:** Material properties of FGM constituents.

Constants	Aluminum	Zirconia(ZrO_2_)	Steel(SUS304)	Silicon(Si3Ni_4_)
*E*, GPa	70.0	151.0	201.04	348.43
*ν*	0.3	0.3	0.3262	0.24
*ρ*, kg/m^3^	2707	3000	8166	2370
*κ*, W/mK	204.0	2.09	12.04	9.19
*c*, J/kgK	896.0	274.0	555.11	496.56
*α*× 10^−^^6^, 1/°C	23.0	10.0	12.33	5.87

**Table 2:** Comparisons of nondimensional frequencies for a fully clamped (CCCC) SUS304/Si_3_Ni_4_ functionally graded square plates thermally loaded by the uniform temperature rise, *T*.

*T*	*p*	Source	${\bar{\omega }}_{1}$	${\bar{\omega }}_{2}={\bar{\omega }}_{3}$	${\bar{\omega }}_{4}$	${\bar{\omega }}_{5}$	${\bar{\omega }}_{6}$	${\bar{\omega }}_{7}={\bar{\omega }}_{8}$
300 K	2	Present	4.1677	7.9565	11.1587	13.1533	13.2784	15.6883
		Li et al. [46]	4.1658	7.9389	11.1212	13.0973	13.2234	15.3627
		∆ ^1^,%	0.0449	0.221	0.337	0.427	0.416	2.119
600 K	2	Present	3.7587	7.3978	10.4864	12.4164	12.5484	15.0855
		Li et al. [46]	3.7202	7.301	10.3348	12.2256	12.3563	14.8112
		∆,%	1.035	1.326	1.467	1.560	1.554	1.852
800 K	2	Present	3.3445	6.8162	9.7681	11.6169	11.7545	14.1711
		Li et al. [46]	3.2741	6.6509	9.5192	11.3126	11.4468	13.7907
		∆,%	2.151	2.486	2.614	2.670	2.688	2.759

^1^ $\Delta =\;\left|\omega_{Ref.}-\omega_{FEM}\right|/\omega_{Ref.}\times 100\%.$

**Table 3:** The first ten nondimensional frequencies of simply supported (SSSS) SUS304/Si_3_Ni_4_ functionally graded square plates thermally loaded by the uniform temperature rise, *T *=600 K.

*p*	Source	${\bar{\omega }}_{1}$	${\bar{\omega }}_{2}={\bar{\omega }}_{3}$	${\bar{\omega }}_{4}={\bar{\omega }}_{5}$	${\bar{\omega }}_{6}$	${\bar{\omega }}_{7}$	${\bar{\omega }}_{8}$	${\bar{\omega }}_{9}$	${\bar{\omega }}_{10}$
0.0		4.1740	10.1983	15.1457	15.7639	19.2859	19.3065	21.4208	24.2877
0.2		3.3782	8.2814	12.2937	12.8114	15.6802	15.6970	17.3859	19.7542
0.5		2.2570	7.0289	10.4044	10.8797	13.3194	13.3386	14.7118	16.7821
1	Present	2.4894	6.1445	9.0147	9.5105	11.6424	11.6618	12.7449	14.6637
	Li et al. [46]	2.5511	6.1761	8.7623	9.5119	11.6301			
	∆,%	2.419	0.512	2.881	0.0147	0.1056			
2	Present	2.2199	5.4930	7.9240	8.4946	10.3932	10.4128	11.2017	13.0762
	Li et al. [46]	2.2690	5.4984	7.7231	8.4675	10.3536			
	∆,%	2.163	0.0979	2.601	0.320	0.382			
5	Present	2.0077	4.9736	7.0232	7.6815	9.3911	9.4105	9.9295	11.8002
	Li et al. [46]	2.0433	4.9538	6.8769	7.6257	9.3240			
	∆,%	1.744	0.399	2.128	0.732	0.719			
10	Present	1.9138	4.7435	6.6588	7.3267	8.9576	8.9762	9.4157	11.2552
	Li et al. [46]	1.9323	4.6881	6.4908	7.2191	8.8282			
	∆,%	0.958	1.183	2.588	1.490	1.466			
∞		1.7644	4.3928	6.2440	6.8006	8.3256	8.3435	8.8315	10.4772

**Table 4:** The first ten nondimensional frequencies of fully clamped (CCCC) SUS304/Si_3_Ni_4_ functionally graded square plates thermally loaded by the uniform temperature rise, *T *= 600 K.

*p*	Source	${\bar{\omega }}_{1}$	${\bar{\omega }}_{2}={\bar{\omega }}_{3}$	${\bar{\omega }}_{4}$	${\bar{\omega }}_{5}$	${\bar{\omega }}_{6}$	${\bar{\omega }}_{7}={\bar{\omega }}_{8}$	${\bar{\omega }}_{9}={\bar{\omega }}_{10}$
0.0		7.1540	13.9341	19.7056	23.3259	23.5522	28.3132	28.3297
0.2		5.7864	11.3046	16.0039	18.9544	19.1406	23.0291	23.1370
0.5		4.8856	9.5742	13.5667	16.0738	16.2347	19.5350	19.7144
1	Present	4.2411	8.3332	11.8143	13.9971	14.1410	17.0117	17.2036
	Li et al. [46]	4.2110	8.2429	11.6602	13.7916	13.9366	16.6856	
	∆,%	0.715	1.096	1.321	1.490	1.467	1.955	
2	Present	3.7587	7.3978	10.4864	12.4164	12.5484	15.0855	15.2405
	Li et al. [46]	3.7202	7.3010	10.3348	12.2256	12.3563	14.8112	
	∆,%	1.035	1.326	1.467	1.560	1.554	1.852	
5	Present	3.3769	6.6504	9.4212	11.1452	11.2674	13.5341	13.6255
	Li et al. [46]	3.3267	6.5424	9.2647	10.9594	11.0790	13.2936	
	∆,%	1.508	1.650	1.689	1.695	1.701	1.809	
10	Present	3.2164	6.3386	8.9808	10.6247	10.7418	12.9026	12.9716
	Li et al. [46]	3.1398	6.1857	8.7653	10.3727	10.4866	12.5971	
	∆,%	2.439	2.472	2.458	2.429	2.434	2.425	
∞		2.9706	5.8867	8.3613	9.9088	10.0175	12.0478	12.2222

**Table 5:** The first ten nondimensional frequencies of SSSS SUS304/Si_3_Ni_4_ functionally graded square plates thermally loaded by the nonlinear temperature rise as shown in Figure 5.

*p*	Source	${\bar{\omega }}_{1}$	${\bar{\omega }}_{2}={\bar{\omega }}_{3}$	${\bar{\omega }}_{4}={\bar{\omega }}_{5}$	${\bar{\omega }}_{6}$	${\bar{\omega }}_{7}$	${\bar{\omega }}_{8}$	${\bar{\omega }}_{9}$	${\bar{\omega }}_{10}$
0.0		4.3081	10.4080	15.3395	16.0514	19.6133	19.6228	21.6944	24.6815
0.2		3.5108	8.4961	12.4954	13.1047	16.0239	16.0313	17.6979	20.1604
0.5		2.9890	7.2447	10.6113	11.1745	13.6691	13.6756	15.0485	17.1928
1	Present	2.6193	6.3551	9.2251	9.7984	11.9851	11.9916	13.0976	15.0665
	Li et al. [46]	2.6576	6.3764	8.9707	9.7992	11.9555			
	∆,%	1.443	0.334	2.836	0.008	0.247			
2	Present	2.3494	5.7002	8.1383	8.7776	10.7309	10.7370	11.5706	13.4738
	Li et al. [46]	2.3727	5.6933	7.9300	8.7468	10.6709			
	∆,%	0.983	0.121	2.627	0.352	0.562			
5	Present	2.1423	5.1904	7.2430	7.9747	9.7453	9.7483	10.3212	12.2111
	Li et al. [46]	2.1424	5.1419	7.0806	7.8970	9.6331			
	∆,%	0.002	0.943	2.293	0.983	1.165			
10	Present	2.0556	4.9764	6.8823	7.6380	9.3385	9.3403	9.8304	11.6898
	Li et al. [46]	2.0465	4.9106	6.7230	7.5386	9.1945			
	∆,%	0.442	1.340	2.369	1.319	1.566			
∞		1.8773	4.5577	6.4224	7.0240	8.5796	8.5865	9.0844	10.7833

**Table 6:** The first ten nondimensional frequencies of CCCC SUS304/Si_3_Ni_4_ functionally graded square plates thermally loaded by the nonlinear temperature rise as shown in Figure 5.

*p*	Source	${\bar{\omega }}_{1}$	${\bar{\omega }}_{2}={\bar{\omega }}_{3}$	${\bar{\omega }}_{4}$	${\bar{\omega }}_{5}$	${\bar{\omega }}_{6}$	${\bar{\omega }}_{7}={\bar{\omega }}_{8}$	${\bar{\omega }}_{9}={\bar{\omega }}_{10}$
0.0		7.3941	14.2773	20.1281	23.7940	24.0181	28.6535	28.8629
0.2		6.0126	11.6262	16.3992	19.3921	19.5759	23.4467	23.5288
0.5		5.1077	9.8879	13.9513	16.4991	16.6573	19.9997	20.0263
1	Present	4.4642	8.6455	12.1960	14.4187	14.5594	17.4727	17.5021
	Li et al. [46]	4.4904	8.6443	12.1559	14.3412	14.4836	17.0433	
	∆,%	0.584	0.0137	0.330	0.540	0.523	2.520	
2	Present	3.9845	7.7113	10.8685	12.8383	12.9667	15.5080	15.5692
	Li et al. [46]	3.9965	7.6961	10.8220	12.7653	12.8934	15.1611	
	∆,%	0.302	0.198	0.429	0.572	0.568	2.288	
5	Present	3.6006	6.9592	9.7974	11.5608	11.6791	13.8901	14.0082
	Li et al. [46]	3.5941	6.9264	9.7400	11.4873	11.6043	13.6331	
	∆,%	0.182	0.473	0.589	0.640	0.644	1.885	
10	Present	3.4343	6.6389	9.3464	11.0290	11.1421	13.2356	13.3630
	Li et al. [46]	3.4243	6.6002	9.2799	10.9425	11.0551	12.9958	
	∆,%	0.291	0.586	0.717	0.790	0.787	1.845	
∞		3.1868	6.1832	8.7213	10.3062	10.4111	12.4894	12.4986

**Table 7:** The first ten nondimensional frequencies of SSSS SUS304/Si_3_Ni_4_ functionally graded square 1-8-1 sandwich plates thermally loaded by the nonlinear temperature rise.

$T_{t}$	*p*		${\tilde{\omega }}_{1}(∆,\%)$	${\tilde{\omega }}_{2}={\tilde{\omega }}_{3}(∆,\%)$	${\tilde{\omega }}_{4}={\tilde{\omega }}_{5}$	${\tilde{\omega }}_{6}$	${\tilde{\omega }}_{7}$	${\tilde{\omega }}_{8}$	${\tilde{\omega }}_{9}$	${\tilde{\omega }}_{10}$
400 K	0.2		9.4089(4.33)	22.620(6.01)	33.590	34.859	42.580	42.588	47.489	53.578
		[39]	9.0180	21.3380						
	0.5		8.4056(4.31)	20.212(5.71)	29.665	31.142	38.039	38.046	41.958	47.854
		[39]	8.058	19.119						
	2		6.9779(3.28)	16.750(4.58)	23.767	25.750	31.415	31.423	33.636	39.449
		[39]	6.756	16.016						
	10		6.2364(2.66)	14.934(3.87)	20.588	22.907	27.910	27.916	29.127	34.984
		[39]	6.075	14.378						
600 K	0.2		9.2157(5.82)	22.320(6.73)	33.298	34.447	42.118	42.142	47.105	53.018
		[39]	8.709	20.912						
	0.5		8.2261(5.65)	19.947(6.57)	29.423	30.783	37.646	37.668	41.671	47.377
		[39]	7.786	18.717						
	2		6.8079(4.34)	16.519(5.20)	23.589	25.447	31.099	31.121	33.484	39.065
		[39]	6.525	15.703						
	10		6.0752(3.74)	14.711(4.33)	20.394	22.608	27.592	27.609	28.924	34.593
		[39]	5.856	14.101						
800 K	0.2		9.0275	22.035	33.030	34.058	41.687	41.727	46.761	52.496
	0.5		8.0516	19.693	29.188	30.439	37.275	37.312	41.412	46.923
	2		6.6437	16.292	23.381	25.140	30.785	30.818	33.336	38.672
	10		5.9216	14.479	20.111	22.275	27.244	27.255	28.691	34.134

**Table 8:** The first ten nondimensional frequencies of CCCC SUS304/Si_3_Ni_4_ functionally graded square 1-8-1 sandwich plates thermally loaded by the nonlinear temperature rise.

$T_{t}$	*p*		${\tilde{\omega }}_{1}\;(∆,\%)$	${\tilde{\omega }}_{2}=\omega_{3}(∆,\%)$	${\tilde{\omega }}_{4}$	${\tilde{\omega }}_{5}$	${\tilde{\omega }}_{6}$	${\tilde{\omega }}_{7}={\tilde{\omega }}_{8}$	${\tilde{\omega }}_{9}={\tilde{\omega }}_{10}$
400 K	0.2		16.213(5.30)	31.190(7.63)	43.912	51.894	52.379	62.855	63.103
		[39]	15.397	28.977					
	0.5		14.460(5.20)	27.809(7.55)	39.144	46.248	46.683	55.893	56.110
		[39]	13.746	25.856					
	2		11.929(3.99)	22.866(6.29)	32.118	37.881	38.248	45.286	45.880
		[39]	11.471	21.513					
	10		10.606(3.26)	20.265(5.59)	28.407	33.450	33.780	39.590	40.461
		[39]	10.271	19.192					
600 K	0.2		15.841(4.23)	30.664(7.22)	43.268	51.182	51.671	62.078	62.543
		[39]	15.199	28.598					
	0.5		14.102(3.73)	27.311(6.83)	38.541	45.586	46.025	55.278	55.519
		[39]	13.594	25.566					
	2		11.566(1.62)	22.381(4.85)	31.545	37.258	37.631	44.921	45.195
		[39]	11.382	21.346					
	10		10.245(0.35)	19.783(3.63)	27.836	32.829	33.166	39.261	39.770
		[39]	10.210	19.09					
800 K	0.2		15.456	30.134	42.629	50.481	50.975	61.298	62.051
	0.5		13.723	26.797	37.924	44.911	45.356	54.543	55.051
	2		11.162	21.844	30.905	36.559	36.940	44.364	44.546
	10		9.817	19.196	27.124	32.042	32.387	38.748	38.886

**Table 9:** The first nine nondimensional frequencies of two edges fully supported and two edges clamped (SCSC) SUS304/Si_3_Ni_4_ functionally graded square 1-8-1 sandwich plates thermally loaded by the nonlinear temperature rise.

$T_{t}$	*p*		${\tilde{\omega }}_{1}(∆,\%)$	${\tilde{\omega }}_{2}(∆,\%)$	${\tilde{\omega }}_{3}$	${\tilde{\omega }}_{4}$	${\tilde{\omega }}_{5}$	${\tilde{\omega }}_{6}$	${\tilde{\omega }}_{7}$	${\tilde{\omega }}_{8}$	${\tilde{\omega }}_{9}$
400 K	0.2		13.274(5.31)	24.522(6.55)	29.754	33.585	39.655	43.561	51.341	56.518	58.691
		[39]	12.605	23.014							
	0.5		11.846(5.25)	21.896(6.49)	26.537	29.655	35.380	38.897	45.768	50.435	51.944
		[39]	11.255	20.560							
	2		9.7941(4.16)	18.105(5.34)	21.845	23.750	29.118	32.087	37.519	41.460	41.810
		[39]	9.403	17.187							
	10		8.7226(3.53)	16.116(4.65)	19.375	20.581	25.813	28.490	33.147	36.370	36.700
		[39]	8.425 15.400								
600 K	0.2		13.010(5.31)	24.201(7.36)	29.301	33.281	39.153	43.093	50.670	55.896	58.203
		[39]	12.354	22.542							
	0.5		11.597(4.99)	21.604(7.13)	26.118	29.388	34.922	38.481	45.185	49.877	51.524
		[39]	11.046	20.166							
	2		9.5510(3.31)	17.840(5.62)	21.451	23.532	28.704	31.729	36.992	40.973	41.485
		[39]	9.245 16.89								
	10		8.4845(2.35)	15.855(4.68)	18.987	20.364	25.403	28.133	32.623	36.050	36.214
		[39]	8.290	15.146							
800 K	0.2		12.742	23.890	28.852	32.999	38.665	42.652	50.077	55.304	57.758
	0.5		11.338	21.313	25.691	29.124	34.464	38.077	44.598	49.329	51.116
	2		9.2850	17.555	21.019	23.267	28.250	31.343	36.406	40.437	41.093
	10		8.2056	15.542	18.514	20.039	24.892	27.683	31.953	35.550	35.593

## References

[1] Amraei M., Shahravi M., Noori Z., Lenjani A. (2014). Application of aluminium honeycomb sandwich panel as an energy absorber of high-speed train nose. J. Compos. Mater..

[2] Manalo A., Aravinthan T., Fam A.Z., Benmokrane B. (2017). State-of-the-art review on FRP sandwich systems for lightweight civil infrastructure. J. Compos. Construct..

[3] Han B., Zhang Z.J., Zhang Q.C., Zhang Q., Lu T.J., Lu B.H. (2017). Recent advances in hybrid lattice-cored sandwiches for enhanced multifunctional performance. Mater. Des..

[4] Altenbach H., Altenbach J., Kissing W. (2018). Mechanics of Composite Structural Elements.

[5] Lu C., Zhao M., Jie L., Wang J., Gao Y., Cui X., Chen P. (2015). Stress distribution on composite honeycomb sandwich structure suffered from bending load. Procedia Eng..

[6] Szekrényes A. (2019). Analytical solution of some delamination scenarios in thick structural sandwich plates. J. Sandw. Struct. Mater..

[7] Burlayenko V.N., Sadowski T., Pietras D. (2018). A numerical analysis of near tip fields in a bending moment-loaded double cantilever sandwich beam fracture specimen. Bull. NTU “KhPI”. Ser. Math. 445 Model. Eng. Technol..

[8] Shi S.S., Sun Z., Hu X.Z., Chen H.R. (2017). Carbon-fiber and aluminum-honeycomb sandwich composites with and without Kevlar-fiber interfacial toughening. Compos. Part A Appl. Sci. Manuf..

[9] Burlayenko V.N., Pietras D., Sadowski T. (2019). Influence of geometry, elasticity properties and boundary conditions on the Mode I purity in sandwich composites. Compos. Struct..

[10] Magnucki K., Jasion P., Szyc W., Smyczynski M.J. (2014). Strength and buckling of a sandwich beam with thin binding layers between faces and a metal foam core. Steel Compos. Struct..

[11] Juhász Z., Szekrényes A. (2017). The effect of delamination on the critical buckling force of composite plates: Experiment and simulation. Compos. Struct..

[12] Burlayenko V.N., Sadowski T., De Roeck G., Degrande G., Lombaert G., Muller G. Numerical modeling of sandwich plates with partially dedonded skin-to-core interface for damage detection. Proceedings of the 8th International Conference on Structural Dynamics EURODYN.

[13] Burlayenko V.N., Sadowski T., Altenbach H., Eremeyev V.A. (2011). Dynamic analysis of debonded sandwich plates with flexible core -Numerical aspects and simulation. Shell-like Structures.

[14] Jayatilake I.N., Karunasena W., Lokuge W. (2016). Finite element based dynamic analysis of multilayer fibre composite sandwich plates with interlayer delaminations. Adv. Aircr. Spacecr. Sci. Int. J..

[15] Savin-Barcan M., Beznea E.-F., Chirica I. (2018). Influence of fabrication imperfections on dynamic response of a sandwich composite panel of a ship deck structure. IOP Conf. Ser. Mater. Sci. Eng..

[16] Burlayenko V.N., Sadowski T. (2014). Transient dynamic response of debonded sandwich plates predicted with finite element analysis. Meccanica.

[17] Qu Y., Meng G. (2017). Nonlinear vibro-acoustic analysis of composite sandwich plates with skin-core debondings. AIAA J..

[18] Idriss M., El Mahi A. (2017). Effects of debonding length on the fatigue and vibration behaviour of sandwich composite. J. Compos. Mater..

[19] Burlayenko V.N., Sadowski T. (2018). Linear and nonlinear dynamic analyses of sandwich panels with face sheet-to-core debonding. Shock Vib..

[20] Burlayenko V.N., Sadowski T. (2014). Simulations of post-impact skin/core debond growth in sandwich plates 480 under impulsive loading. J. Appl. Nonlin. Dyn..

[21] Funari M.F., Greco F., Lonetti P. (2018). Sandwich panels under interfacial debonding mechanisms. Compos. Struct..

[22] Burlayenko V.N., Altenbach H., Sadowski T., Altenbach H., Belyaev A., Eremeyev V.A., Krivtsov A., Porubov A.V. (2019). Dynamic fracture analysis of sandwich composites with face sheet/core debond by the finite element method. Dynamical Processes in Generalized Continua and Structures.

[23] Zenkour A.M. (2005). A comprehensive analysis of functionally graded sandwich plates: part 1-Deflection and 488 stresses. Int. J. Solids Struct..

[24] Kanu N.J., Vates U.K., Singh G.K., Chavan S. (2019). Fracture problems, vibration, buckling, and bending analyses of functionally graded materials: A state-of-the-art review including smart FGMS. Particul. Sci. Technol..

[25] Ghazaryan D., Burlayenko V.N., Avetisyan A., Bhaskar A. (2018). Free vibration analysis of functionally graded beams with non-uniform cross-section using the differential transform method. J. Eng. Math..

[26] Li C., Shen H.-S., Wang H. Nonlinear dynamic response of sandwich beams with functionally graded negative Poisson’s ratio honeycomb core. Eur. Phys. J. Plus.

[27] Vakili-TAHAMI F., Mahkam N., Fard A.M.A. (2017). Optimum design of functionally graded plates under thermal 499 shock. UPB Sci. Bull. Ser. D Mechan. Eng..

[28] Do T.V., Bui T.Q., Yu T.T., Pham D.T., Nguyen C.T. (2017). Role of material combination and new results of mechanical behaviorfor FG sandwich plates in thermal environment. J. Comput. Sci..

[29] Petrova V., Schmauder S. (2017). Modeling of thermo-mechanical fracture of FGMs with respect to multiple cracks 504 interaction. Phys. Mesomech..

[30] Burlayenko V.N. (2016). Modelling thermal shock in functionally graded plates with finite element method. Adv. Mater. Sci. Eng..

[31] Pathak H. (2017). Three-dimensional quasi-static fatigue crack growth analysis in functionally graded materials (FGMs) using coupled FE-XEFG approach. Theor. Appl. Fract. Mec..

[32] Ivanov I., Velchev D., Penkova N., Krumov K., Iliev V. (2018). Stress analysis of insulating glass units under transient thermal loadings. J. Chem. Technol. Metall..

[33] Zhang H.H., Han S.Y., Fan L.F., Huang D. (2018). The numerical manifold method for 2D transient heat conduction problems in functionally graded materials. Eng. Anal. Bound. Elem..

[34] Swaminathan K., Sangeetha D.M. (2017). Thermal analysis of FGM plates-A critical review of various modeling techniques and solution methods. Compos. Struct..

[35] Reddy J.N. (2000). Analysis of functionally graded plated. Int. J. Numer. Meth. Eng..

[36] Matsunaga H. (2007). Free vibration and stability of angle-ply laminated composite and sandwich plates under 520 thermal loading. Compos. Struct..

[37] Zenkour A.M. (2009). The effect of transverse shear and normal deformations on the thermomechanical bending of functionally graded sandwich plates. Int. J. Appl. Mech..

[38] Fazzolari F.A. (2015). Natural frequencies and critical temperatures of functionally graded sandwich plates subjected to uniform and non-uniform temperature distributions. Compos. Struct..

[39] Pandey S., Pradyumna S. (2015). Free vibration of functionally graded sandwich plates in thermal environment using a layerwise theory. Eur. J. Mech. A Solid.

[40] Mantari J.L., Granados E.V. (2015). Thermoelastic behavior of advanced composite sandwich plates by using a new 6 unknown quasi-3D hybrid type HSDT. Compos. Struct..

[41] Do V.N.V., Lee C.-H. (2018). Quasi-3D higher-order shear deformation theory for thermal buckling analysis of FGM plates based on a meshless method. Aerosp. Sci. Technol..

[42] Han B., Hui W.-W., Zhang Q.-C., Zhao Z.-Y., Jin F., Zhang Q., Lu T.J., Lu B.-H. (2018). A refined quasi-3D zigzag beam theory for free vibration and stability analysis of multilayered composite beams subjected to thermomechanical loading. Compos. Struct..

[43] Reddy J.N., Cheng Z.-Q. (2003). Frequency of functionally graded plates with three-dimensional asymptotic approach. J. Eng. Mech..

[44] Reddy J.N., Chen Z.-Q. (2001). Three-dimensional thermomechanical deformations of functionally graded rectangular plates. Eur. J. Mech. A Solids.

[45] Vel S.S., Batra R.C. (2003). Three-dimensional analysis of transient thermal stresses in functionally graded plates. Int. J. Solids Struct..

[46] Li Q., Iu V.P., Kou K.P. (2009). Three-dimensional vibration analysis of functionally graded material plates in thermal environment. J. Sound Vibr..

[47] Alibeigloo A. (2010). Exact solution for thermo-elastic response of functionally graded rectangular plates. Compos. Struct..

[48] Brischetto S., Torre R. (2019). 3D shell model for the thermo-mechanical analysis of FGM structures via imposed and calculated temperature profiles. Aerosp. Sci. Technol..

[49] Zhang Z. (2007). (Jenny); Paulino, G.H. Wave propagation and dynamic analysis of smoothly graded heterogeneous continua using graded finite elements. Int. J. Solids Struct..

[50] Asemi K., Salehi M., Akhlaghi M. (2015). Three dimensional graded finite element elasticity shear buckling analysis of FGM annular sector plates. Aerosp. Sci. Technol..

[51] Santare M.H., Thamburaj P., Gazonas G.A. (2003). The use of graded finite elements in the study of elastic wave propagation in continuously nonhomogeneous materials. Int. J. Solids Struct..

[52] Brischetto S., Carrera E. (2010). Coupled thermo-mechanical analysis of one-layered and multilayered plates. Compos. Struct..

[53] Bui T.Q., Do T.V., Ton L.H.T., Doan D.H., Tanaka S., Pham D.T., Nguyen-Van T.-A., Yu T., Hirose S. (2016). On the high temperature mechanical behaviors analysis of heated functionally graded plates using FEM and a new third-order shear deformation plate theory. Compos. Part B Eng..

[54] Pandey S., Pradyumna S. (2018). Transient stress analysis of sandwich plate and shell panels with functionally graded material core under thermal shock. J. Therm. Stresses.

[55] Moleiro F., Franco Correia V.M., Ferreira A.J.M., Reddy J.N. (2019). Fully coupled thermo-mechanical analysis of multilayered plates with embedded FGM skins or core layers using a layerwise mixed model. Compos. Struct..

[56] Dassault Systémes Simulia Corp (2016). ABAQUS User’s Manual Ver. 2016.

[57] Reinoso J., Blázquez A. (2016). Geometrically nonlinear analysis of functionally graded power-based and carbon nanotubes reinforced composites using a fully integrated solid shell element. Compos. Struct..

[58] Buttlar W.G., Paulino G.H., Song S.H. (2006). Application of graded finite elements for asphalt pavements. J. Eng. 577 Mech..

[59] Burlayenko V.N., Altenbach H., Sadowski T., Dimitrova S.D., Bhaskar A. (2017). Modelling functionally graded materials in heat transfer and thermal stress analysis by means of graded finite elements. Appl. Math. Model..

[60] Mars J., Kouba S., Wali M., Dammak F. (2017). Numerical analysis of geometrically non-linear behavior of functionally graded shells. Lat. Am. J. Solids Struct..

[61] Shiyekar S.M., Lavate P. (2015). Flexure of power law governed functionally graded plates using ABAQUS UMAT. Aerosp. Sci. Technol..

[62] Burlayenko V.N., Sadowski T. (2019). Free vibrations and static analysis of functionally graded sandwich plates with three-dimensional finite elements. Meccanica.

[63] Burlayenko V.N., Altenbach H., Sadowski T., Dimitrova S.D., Altenbach H., Chróścielewski J., Eremeyev V.A., Wiśniewski K. (2019). Three-dimensional finite element modelling 589 of free vibrations of functionally graded sandwich panels. Recent Developments in the Theory of Shells.

[64] Hetnarski R.B., Eslami M.R. (2009). Thermal Stresses-Advanced Theory and Applications.

[65] Sutradhar A., Paulino G.H. (2004). The simple boundary element method for transient heat conduction in functionally graded materials. Comput. Methods Appl. Mech. Eng..

[66] Gajewski J., Sadowski T. (2014). Sensitivity analysis of crack propagation in pavement bituminous layered structures using a hybrid system integrating Artificial Neural Networks and Finite Element Method. Comp. Mater. Sci..

[67] Sadowski T., Kneć M., Golewski P. (2014). Experimental investigation and numerical modelling of spot welding-adhesive joints response. Compos. Struct..

